# Efgartigimod for generalized myasthenia gravis in the extreme elderly (≥80 years): a multicenter retrospective real-world study

**DOI:** 10.3389/fimmu.2025.1685233

**Published:** 2025-11-06

**Authors:** Ye Hong, Chang-Wen Yuan, Jie Lu, Ping-Ping Kong, Shi-Qi Huang, Rui-Yuan Yang, Zhen-Hua Yuan, Hong-Dong Zhao, Teng Jiang, Jing Chen, Jian-Quan Shi

**Affiliations:** 1 Department of Neurology, Nanjing First Hospital, Nanjing Medical University, Nanjing, China; 2 Department of Neurology, Jiangsu Province Hospital of Chinese Medicine, Affiliated Hospital of Nanjing University of Chinese Medicine, Nanjing, China; 3 Department of Neurology, The Affiliated Brain Hospital of Nanjing Medical University, Nanjing, China; 4 Department of Neurology and Clinical Research Center of Neurological Disease, The Second Affiliated Hospital of Soochow University, Suzhou, China

**Keywords:** myasthenia gravis, efgartigimod, elderly, VLOMG, MG-ADL, MGAE

## Abstract

**Background:**

Target-specific immunotherapies have been shown to effectively treat myasthenia gravis (MG) with less side effects. One such immunotherapy is efgartigimod, a neonatal Fc receptor antagonist, promotes degradation of pathogenic IgG antibodies. However, data specifically focusing on elderly, especially for those over 80 years, remain limited.

**Methods:**

This study included generalized MG patients over 80 years old from four neuromuscular centers who were treated with efgartigimod. Data regarding MG history, treatment regimens, and scores from the MG-ADL, QMG, and MGC scales, as well as adverse events, were prospectively recorded.

**Results:**

Twelve patients with mean age of 82.9 ± 2.5 years were included. Anti-AChR antibodies were positive in 11 patients and anti-MuSK antibodies were detected in 1 patient. All patients received at least one cycle of efgartigimod treatment, for the following indications: myasthenia gravis acute exacerbation (MGAE, n=8), mild/moderate disease (n=3), and myasthenic crisis (MC, n=1). At week 4 (1week after the final infusion), the study showed significant reductions in all efficacy measurements: MG-ADL scores decreased by 52.2% ± 30.8% (from 10.9 ± 2.7 to 3.6 ± 2.1), QMG scores by 36.6% ± 28.4% (from 18.2 ± 7.0 to 11.7 ± 8.3), MGC scores by 48.2% ± 33.4% (from 17.8 ± 7.5 to 9.0 ± 8.6). The greatest improvement was observed in the MGAE subgroup, with reductions of 69.8% ± 16.9% in MG-ADL, 51.8% ± 20.6% in QMG, 66.5% ± 20.2% in MGC. Clinically meaningful improvement (CMI) was rapidly achieved by 91.7% (11/12) of patients at 1.3 ± 0.5 weeks, with 8.3% (1/12) reaching minimal symptom expression (MSE) by week 4. However, two patients in the mild/moderate group failed to sustain CMI through week 4, resulting in nine responders overall (8 MGAE, 1mild/moderate). These responders maintained symptom control throughout the 24-week follow-up with subsequent therapies. Treatment-related adverse events were mild: two patients experienced transient minor headache and one patient had mild upper respiratory tract infection.

**Conclusions:**

This multicenter study demonstrated that efgartigimod was efficacious and safe in the elderly MG over 80 years of age. Elderly patients with MGAE presented to benefit the most from efgartigimod treatment.

## 1 Introduction

Myasthenia gravis (MG) is an autoimmune disease triggered by various pathogenic immunoglobulin G (IgG) autoantibodies disrupting the neuromuscular junction, leading to the fluctuant of muscular weakness (1, 2). Based on differences in clinical and therapeutic features, MG patients are classified into three age subgroups: early-onset MG (EOMG, age at onset < 50 years), late-onset MG (LOMG, onset age ≥50 and <65 years), and very-late-onset MG (VLOMG, onset age ≥65 years) (3). Recently epidemiological studies indicated a growing number of MG patients worldwide, primarily due to increasing prevalence and incidence rate in the LOMG and VLOMG (4–6). Consequently, elderly MG patients have garnered increased clinical attention.

Treating elderly MG patients is challenging for several reasons. First, MG management is inherently difficult. Traditionally used corticosteroids and non-specific immunotherapies have exhibit variable efficacy and carry significant side-effect burdens (7). Despite therapeutic advances, approximately 10-20% of MG patients experienced myasthenic crisis (MC) in their life (8). Second, the elderly is a vulnerable population with more comorbidities and a higher probability to present with treatment associated adverse effects. A multicentric retrospective study from France highlighted substantial iatrogenic risks, including fatal immunosuppressant-related infections, in the elderly MG (9). Third, the pathogenesis of MG in elderly may be different from that in the younger patients. For example, EOMG has a female predominance while LOMG occurs slightly more frequently in men (3, 5). The concentration of AChR antibodies was lower in the elderly MG, and thymectomy has been confirmed to offer no benefit in non-thymomatous LOMG cases (10, 11). Finally, there is a lack of clinical trials specifically designed to test the efficacy and safety of drugs in elderly MG. Although the few available studies have demonstrated a good prognosis of VLOMG with fewer immunosuppressants when diagnosed and treated properly, managing elderly MG remains clinically challenging (3, 5, 9, 11).

Target-specific immunotherapies have been shown to effectively treat MG with fewer side effects. Efgartigimod is a first-in-class neonatal Fc receptor (FcRn) blocker approved for the treatment of AChR antibody-positive generalized MG (gMG) in the USA, Japan, and the Europe, based on the phase 3 ADAPT clinical trial (12). In 2023, efgartigimod was also approved in China as an add-on therapy for adult AChR antibody-positive gMG patients. Real-world studies have further shown that a wide spectrum of MG patients, including those with MuSK-antibody positivity, triple-seronegativity, and even myasthenic crisis, respond rapidly to efgartigimod with a favorable safety profile (13–16). However, the clinical experiences with efgartigimod in elderly gMG patients, especially those with very-late onset, remains sparse. The average age of patients in the efgartigimod treatment group of the ADAPT trial was 45.9 ± 14.4, with the oldest participant being 78 years old (12). While some real-world studies included relatively older patients than the ADAPT trial, most did not perform age-stratified analyses (13, 14). Thus, data on the efficacy and safety of efgartigimod in MG patients aged 80 years or older are lacking.

In this multicenter study, we aimed to evaluate the effects of efgartigimod in the elderly MG patients aged 80 years or older. We hypothesized that efgartigimod would provide rapid disease control in this elderly population with a favorable safety profile.

## 2 Methods

### 2.1 Patients

This observational multicenter study was conducted in the neuromuscular centers of 4 hospitals in Jiangsu Province, China. These hospitals were Nanjing First Hospital, The Second Affiliated Hospital of Soochow University, Jiangsu Province Hospital of Chinese Medicine, and Nanjing Brain Hospital. All consecutive MG patients aged ≥80 years who received at least one infusion of efgartigimod between October 1, 2023 to November 30, 2024 across the four participating centers were included. MG diagnosis was established by clinical presentations in accordance with gMG and positive with AChR or MuSK antibodies, or seronegative but positive with repetitive nerve stimulation (RNS). Chest computed tomography (CT) was performed in all patients to investigate thymus. None of these patients had received treatment with FcRn blocker before. This study was approved by the Ethics Committee of Nanjing First Hospital in compliance with the Declaration of Helsinki. Written informed consent was obtained from all study participants.

### 2.2 Clinical data collection

Information about demographic features and disease-associated variables, including sex, age, comorbidities, eGFR, date of onset, disease duration, antibody status, history of thymoma and thymectomy, disease severity at onset and admission, clinical classification, myasthenia gravis activities of daily living score (MG-ADL), Myasthenia Gravis Composite score (MGC), quantitative Myasthenia Gravis score (QMG), MG specific therapy at the time of efgartigimod initiation, and the indications to use efgartigimod as an add-on therapy were extracted from the medical files. The disease severity was evaluated by the Myasthenia Gravis Foundation of America (MGFA) clinical classification. Classification of MG was conducted according to national guidelines. EOMG was non-thymomatous AChR antibody-positive generalized MG with onset before age 50 years (3). LOMG was non-thymomatous AChR antibody-positive generalized MG with onset after age 50 years and < 65 years. VLOMG was non-thymomatous AChR antibody-positive gMG with onset age ≥65 years. TMG was gMG with thymoma regardless of onset ages. MuSK-MG was MuSK antibody-positive gMG. SNMG referred to generalized MG patients who were double-negative with AChR and MuSK antibodies. MG acute exacerbation (MGAE) was defined as progressive clinical deterioration induced by weakness of the bulbo-pharyngeal or limb muscles or decreased respiratory function affecting daily activities within 30 days (8). Myasthenic crisis (MC) referred to serious, life-threatening, rapid clinical decline that requires noninvasive ventilation or intubation with mechanical ventilation.

Eleven patients completed the first cycle of efgartigimod (weekly infusion of 10mg/Kg efgartigimod for four consecutive weeks). One patient received three of four infusions of the first cycle of efgartigimod and further infusion was refused due to excellent clinical symptom control by the three infusions. Additionally, 5 patients received a second cycle of efgartigimod with variable dosing intervals (weekly to monthly). Patients’ IgG levels were measured before and at 1 week after the final injection of efgartigimod. The main treatment outcome was evaluated by MG-ADL, MGC and QMG. After efgartigimod administration, MG-ADL, MGC and QMG score, treatment adverse effects (TAE), and any changes of prednisone dose and other immunotherapies were prospectively recorded for 24 weeks after the first infusion of efgartigimod treatment. Clinical meaningful improvement (CMI) in MG-ADL scores was defined as reduction of ≥2 points from baseline values. CMI in MGC and QMG scores were both defined as reduction of ≥3 points from baseline values. Minimal symptom expression (MSE) was defined as an MG-ADL score of 0 or 1.

### 2.3 Statistical analysis

Statistical analyses were performed with SPSS 22.0 (SPSS Inc., Chicago, IL, USA). Continuous variables were initially assessed for normality, and those conformed to normal distribution were expressed as mean ± standard deviation (SD). Categorical variables were described as frequency (percentage).

## 3 Results

### 3.1 Baseline clinical characteristics

Twelve (12) gMG patients aged over 80 years who received efgartigimod treatment were included from four hospitals. Baseline clinical features are detailed in **Table 1**. The mean onset age was 81.3 ± 4.1 years (range: 74-89), the mean age at admission was 82.9 ± 2.5 years (range: 80–90) and the mean disease duration was 20.6 ± 34.6 months (range: 1–120). A total of 38 comorbidities were recorded for the 12 patients: 16 cardiovascular, 5 gastrointestinal, 5 neurological, 2 respiratory, 2 musculoskeletal, 2 immune-related, and 1 genitourinary. Only 1 (8.3%) patient had normal renal function with eGFR ≥ 90 ml/min_*_1.73m^2^. Eight patients (66.7%) had mild renal impairment (60 ≤ eGFR < 90 ml/min_*_1.73m^2^), and 3 patients (25.0%) had moderate renal impairment (30 ≤ eGFR < 60 ml/min_*_1.73m^2^). All patients had normal liver functions, indicated by serum alanine aminotransferase (ALT) and aspartate aminotransferase (AST) levels within normal ranges.

**Table 1:** Baseline clinical characteristics of 12 elderly gMG patients (age>80 years) who were treated with efgartigimod.

Clinical variables	Mean ± SD (range) or No. (%)
Age (years old)	82.9 ± 2.5
Sex (Male %)	2 (16.7%)
Comorbidities	
Respiratory system disease	2
Gastrointestinal system disease	5
Genitourinary system disease	1
Musculoskeletal system disease	2
Nervous system disease	5
Cardiovascular system disease	16
Immune system disease	2
Endocrine system disease	5
Thymoma (%)	0 (0%)
Thymectomy (%)	0 (0%)
Renal function (%)	
eGFR^*^ ≥ 90	1 (8.3%)
60 ≤ eGFR < 90	8 (66.7%)
30 ≤ eGFR < 60	3 (25.0%)
15 ≤ eGFR < 30	0 (0%)
eGFR < 15	0 (0%)
Onset age (years)	81.3 ± 4.1
Disease duration (months)	20.6 ± 34.6
MGFA classification at onset	
I	6 (50.0%)
II	4 (33.3%)
III	0 (0%)
IV	2 (16.7%)
V	0 (0%)
Clinical classification with efgartigimod initiation	
EOMG	0 (0%)
LOMG	0 (0%)
VLOMG	11 (91.7%)
TMG	0 (0%)
MUSK-MG	1 (8.3%)
SNMG	0 (0%)
Previous treatment	
Pyridostigmine	11 (91.7%)
Prednisone	7 (58.3%)
Tacrolimus	1 (8.3%)
Azathioprine	0 (0%)
Mycophenolate mofetil	1 (8.3%)
Cyclophosphamide	0 (0%)
Rituximab	0 (0%)
Long-term sustained IVIg/PE	0 (0%)
MGFA classification with efgartigimod initiation	
I	0 (0%)
II	5 (41.7%)
III	5 (41.7%)
IV	1 (8.3%)
V	1 (8.3%)
Initiation status	
MGAE	8 (66.7%)
MC	1 (8.3%)
Mild/moderate	3 (25%)
Prednisone dosage at baseline (mg/d)	10.8 ± 9.7

EOMG, early-onset MG; gMG: generalized myasthenia gravis; IVIg, intravenous immunoglobulin; LOMG, late-onset MG; MC, myasthenic crisis.; MGAE, MG acute exacerbation; MGFA, Myasthenia Gravis Foundation of America; MuSK, muscle-specific tyrosine kinase; PE, plasma exchange; SNMG, seronegative MG; TMG, thymoma-associated MG. * The unite of eGFR is ml/min_*_1.73m^2^.

This multicenter cohort included 11 AChR-MG (91.7%), 1 (8.3%) MuSK-MG, but no TMG or SNMG patients. All patients were VLOMG. CT examination of thymus was normal for all patients, and none received thymectomy. MGFA classes at disease onset were: MGFA I (50.0%, 6/12), MGFA II (33.3%, 4 o/12), MGFA III (0%), MGFA IV (16.7%, 2/12), and MGFA V (0%). Before efgartigimod initiation, pyridostigmine was employed in 91.7% (11/12) of patients. Immunosuppressants (IS) including glucocorticoids (66.7%, 8/12), tacrolimus (8.3%, 1/12), and mycophenolate mofetil (8.3%, 1/12), no patients used azathioprine, cyclophosphamide, IVIg, TPE, or rituximab. The prednisone dosage at baseline was 16.3 ± 6.9 mg/d.

The reasons for initiating efgartigimod were highly active disease (75.0%, 9/12), including MGAE (66.7%, 8/12) and MC (8.3%, 1/12), and mild/moderate disease (25%, 3/12). The MGFA classes with efgartigimod initiation were: MGFA II (41.7%, 5/12), MGFA III (41.7%, 5/12), MGFA IV (8.3%, 1/12), and MGFA V (8.3%, 1/12).

### 3.2 Clinical response to the first cycle of efgartigimod in elderly patients

**Figure 1:**
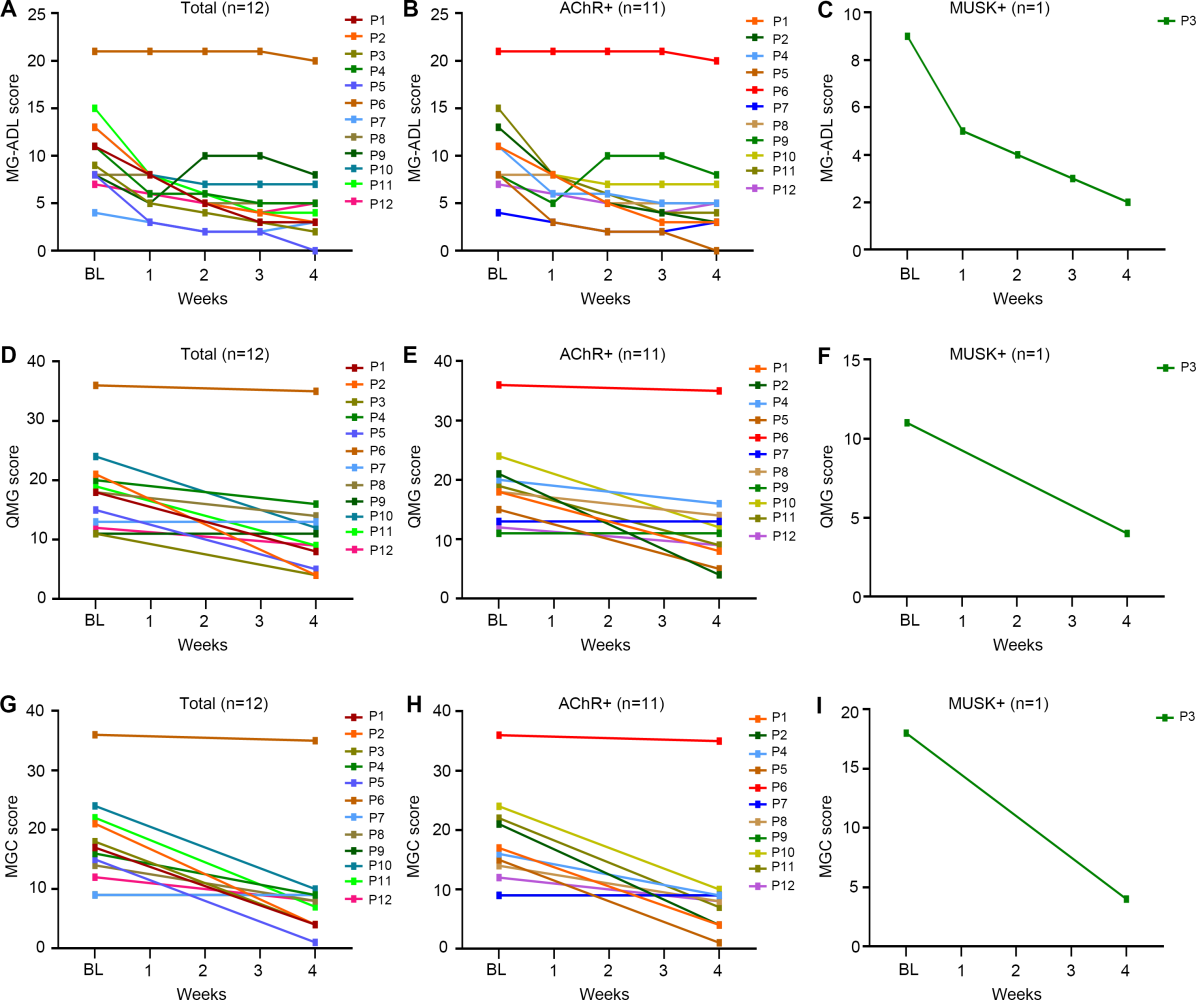
The changes in different clinical scores of the elderly gMG patients stratified by the serotypes of antibody from baseline to week 4 after efgartigimod injection. **(A-C)** Detailed changes of MG-ADL scores; **(D-F)** Detailed changes of QMG scores; **(G-I)** Detailed changes of MGC scores. Each line represented one patient. BL, baseline; MG-ADL, Myasthenia Gravis-Activities of Daily Living; MGC, Myasthenia Gravis Composite; QMG, quantitative Myasthenia Gravis.

All patients received one cycle (four infusions) of efgartigimod, except for one patient (P3) who refused the fourth infusion of efgartigimod due to fast and excellent response after three infusions. The longitudinal changes in MG-ADL, QMG and MGC scores from baseline to week 4 were shown in **Figures 1A, D, G**. Under efgartigimod treatment, CMI was rapidly achieved in 91.7% (11/12) of patients, with a mean time of 1.3 ± 0.5 weeks. However, two patients did not maintain the CMI status until week 4. Only one patient (8.3%) achieved MSE by week 4 (**Table 2**). For all twelve patients, the MG-ADL score decreased from 10.7 ± 4.5 to 5.4 ± 5.1 (a 52.2% ± 30.8% reduction), the QMG score from 18.2 ± 7.0 to 11.7 ± 8.3 (a 36.6% ± 28.4% reduction), the MGC score from 17.8 ± 7.5 to 9.0 ± 8.6 (a 48.2% ± 33.4% reduction) by week 4.

**Table 2:** The efficacy profile of the first cycle of efgartigimod in 12 elderly gMG patients with age>80 years.

Group	MG-ADL						QMG		MGC	
	Time to CMI (w)	Baseline (MSE%)	1w (MSE%)	2w (MSE%)	3w (MSE%)	4w (MSE%)	Baseline	4w	Baseline	4w
All patients (n=12)	1.3 ± 0.5	10.7 ± 4.5 (0%)	7.42 ± 4.7 (0%)	6.5 ± 5.0 (0%)	5.8 ± 5.3 (0%)	5.4 ± 5.1 (8.3%)	18.2 ± 7.0	11.7 ± 8.3	17.8 ± 7.5	9.0 ± 8.6
AChR-Ab+ (n=11)	1.3 ± 0.5	10.8 ± 4.6 (0%)	7.6 ± 4.8 (0%)	6.7 ± 5.2 (0%)	6.1 ± 5.4 (0%)	5.7 ± 5.2 (9.1%)	18.8 ± 7.0	12.4 ± 8.3	17.7 ± 7.8	9.5 ± 8.9
MUSK-Ab+ (n=1)	1.0 ± 0	9.0 ± 0 (0%)	5.0 ± 0 (0%)	4.0 ± 0 (0%)	3.0 ± 0 (0%)	2.0 ± 0 (0%)	11.0 ± 0	4.0 ± 0	18.0 ± 0	4.0 ± 0
Initiation status										
MGAE (n=8)	1.1 ± 0.4	10.9 ± 2.7 (0%)	6.5 ± 1.9 (0%)	5.0 ± 1.5 (0%)	4.0 ± 1.5 (0%)	3.6 ± 2.1 (12.5%)	17.5 ± 4.5	8.4 ± 4.2	18.1 ± 4.0	5.9 ± 3.1
MC (n=1)	6.0 ± 0	21.0 ± 0 (0%)	21.0 ± 0 (0%)	21.0 ± 0 (0%)	21.0 ± 0 (0%)	20.0 ± 0 (0%)	36.0 ± 0	35.0 ± 0	36.0 ± 0	35.0 ± 0
mild/moderate (n=3)	1.7 ± 0.6	6.7 ± 2.3 (0%)	5.3 ± 2.5 (0%)	5.7 ± 4.0 (0%)	5.7 ± 4.0 (0%)	5.3 ± 2.5 (0%)	14.0 ± 3.6	12.7 ± 1.5	10.7 ± 2.9	8.7 ± 0.6

CMI, clinical meaningful improvement; MG-ADL, Myasthenia Gravis-Activities of Daily Living; MGC, Myasthenia Gravis Composite; MSE, minimal symptom expression; MGAE, myasthenia gravis acute exacerbation; MC, myasthenic crisis; QMG, quantitative Myasthenia Gravis.

**Figure 2:**
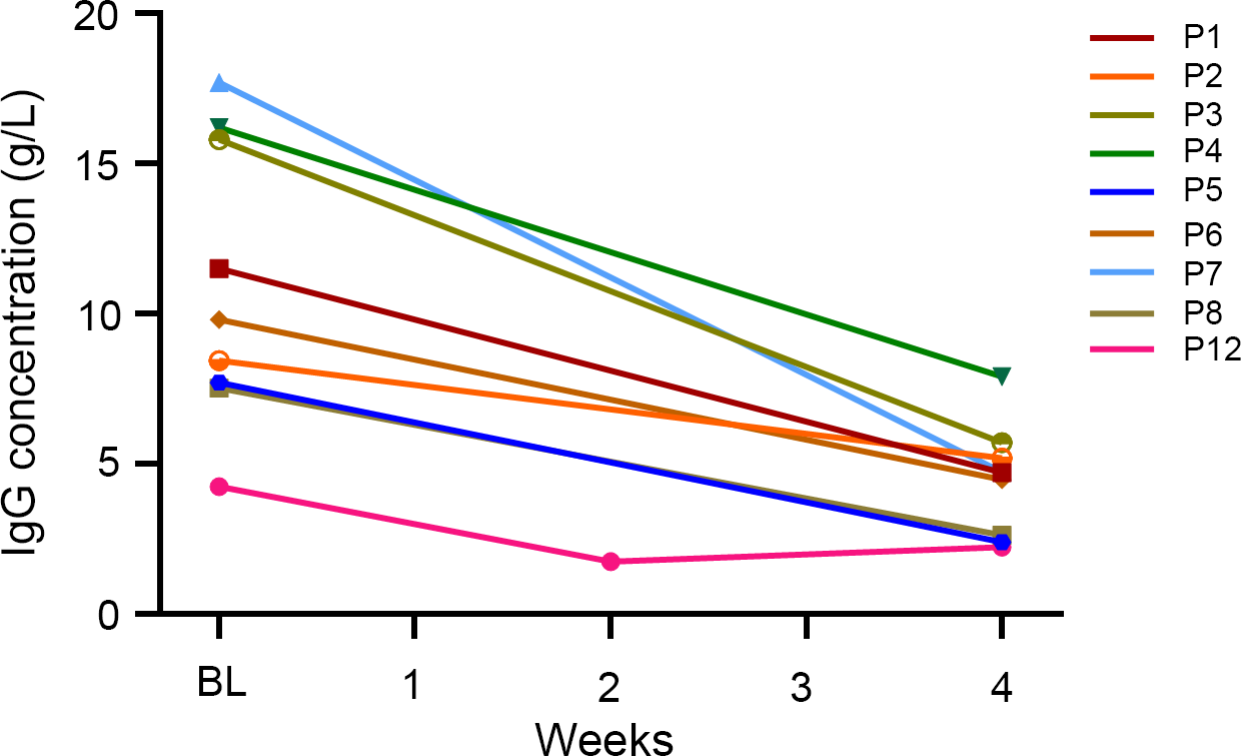
The effect of efgartigimod on serum IgG concentrations (n = 9). Serum IgG concentrations were detected before and at 1-week after the four infusions of efgartigimod. Each line represented one patient. BL, baseline.

IgG levels before and after efgartigimod treatment were available for nine patients. A 58.0% reduction in IgG levels was observed one week after the fourth infusion of efgartigimod (from the baseline 11.0 ± 4.6 g/L to 4.4 ± 1.8 g/L at week 4, **Figure 2**). Notably, the maximum reduction of IgG was observed in P12 one week after the second infusion, decreasing from the baseline 4.2 g/L to 1.74 g/L. As the clinical presentation improved, the remaining two infusions were administrated with weekly IgG monitoring. However, the serum concentration of IgG did not decrease further and returned to 2.2 g/L one week after the fourth infusion.

### 3.3 Subgroup analysis of therapeutic response to efgartigimod in elderly patients

Patients were stratified by clinical subtypes. All AChR-Ab+ patients were VLOMG, there was one MuSK-MG and no SNMG case. As shown in **Table 2**, the AChR-Ab+ patients had relatively higher baseline scores than the MuSK-MG patient (MG-ADL:10.8 *vs* 9.0; QMG: 18.8 *vs* 11.0),and the time to achieved CMI was longer (1.3-week *vs* 1.0-week). A consistent decline in MG-ADL, QMG and MGC were observed in AChR-Ab+ patients during and 1 week after efgartigimod treatment (**Figures 1B, E, H**). By week 4, the MG-ADL score in these AChR-Ab+ patients had decreased from a baseline of 11.3 ± 2.6 to 5.7 ± 5.2, the QMG score from 18.8 ± 7.0 to 12.4 ± 8.3, and the MGC score from 17.7 ± 7.8 to 9.5 ± 8.9 (**Table 2**). Minimal symptom expression (MSE) was achieved in 9.1% (1/11) of AChR-Ab+ patients. In the single MuSK-Ab+ patient, efgartigimod treatment led to a marked improvement by week 4, with reductions in the MG-ADL score (from 9 to 2), QMG score (from 11 to 4), and MGC score (from 18 to 4) (**Table 2**, **Figures 1C, F, I**).

**Figure 3:**
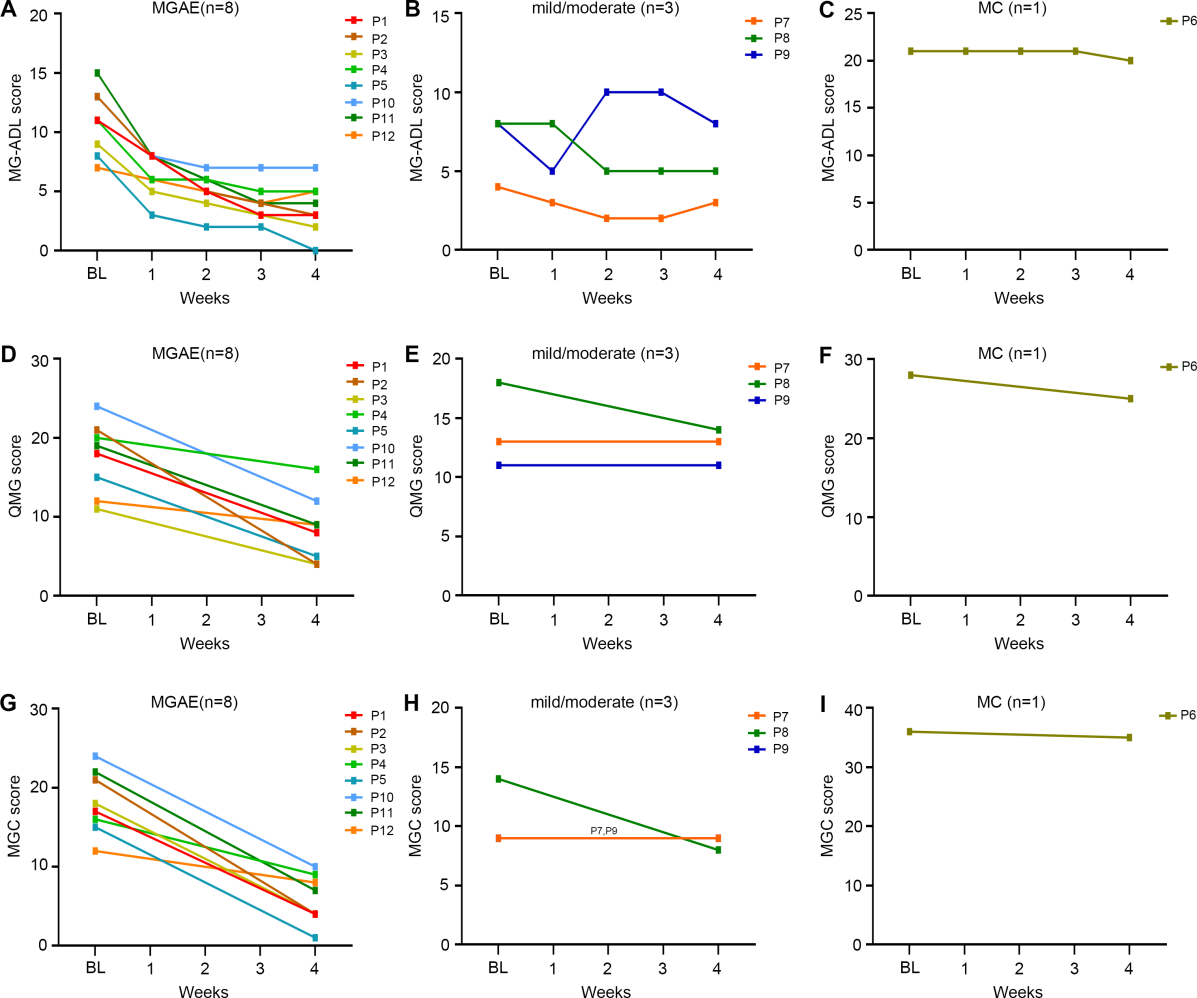
The changes in different clinical scores of the elderly gMG patients stratified by the initiation disease status from baseline to week 4 after efgartigimod injection. **(A-C)** Detailed changes of MG-ADL scores; **(D-F)** Detailed changes of QMG scores; **(G-I)** Detailed changes of MGC scores. Each line represented one patient. BL, baseline; MG-ADL, Myasthenia Gravis-Activities of Daily Living; MGC, Myasthenia Gravis Composite; QMG, quantitative Myasthenia Gravis.

Patients were further stratified according to disease status at treatment initiation. In the MGAE group (n=8), all scores demonstrated significant reductions by week 4: MG-ADL decreased from 10.9 ± 2.7 to 3.6 ± 2.1; QMG from 17.5 ± 4.5 to 8.4 ± 4.2; and MGC from 18.1 ± 4.0 to 5.9 ± 3.1 (**Table 2**). MSE status was achieved by 12.5% (1/8) of patients in this group. More modest improvements were observed in the mild/moderate group (n=3), with the MG-ADL score decreasing from 6.7 ± 2.3 to 5.3 ± 2.5, the QMG from 14.0 ± 3.6 to 12.7 ± 1.5, and the MGC from 10.7 ± 2.9 to 8.7 ± 0.6. The single MC patient showed a minimal response, evidenced by only a 1-point reduction across all scales (MG-ADL: 21.0 to 20.0; QMG: 36.0 to 35.0; MGC: 36.0 to 35.0). Although scores continued decline post-treatment, neither the MC patient nor any mild/moderate cases attained MSE during follow-up period. The change trends of MG-ADL, QMG, and MGC scores for patients with different initiation status were shown in **Figure 3**.

### 3.4 Post-efgartigimod sequential therapies and clinical responses

**Figure 4:**
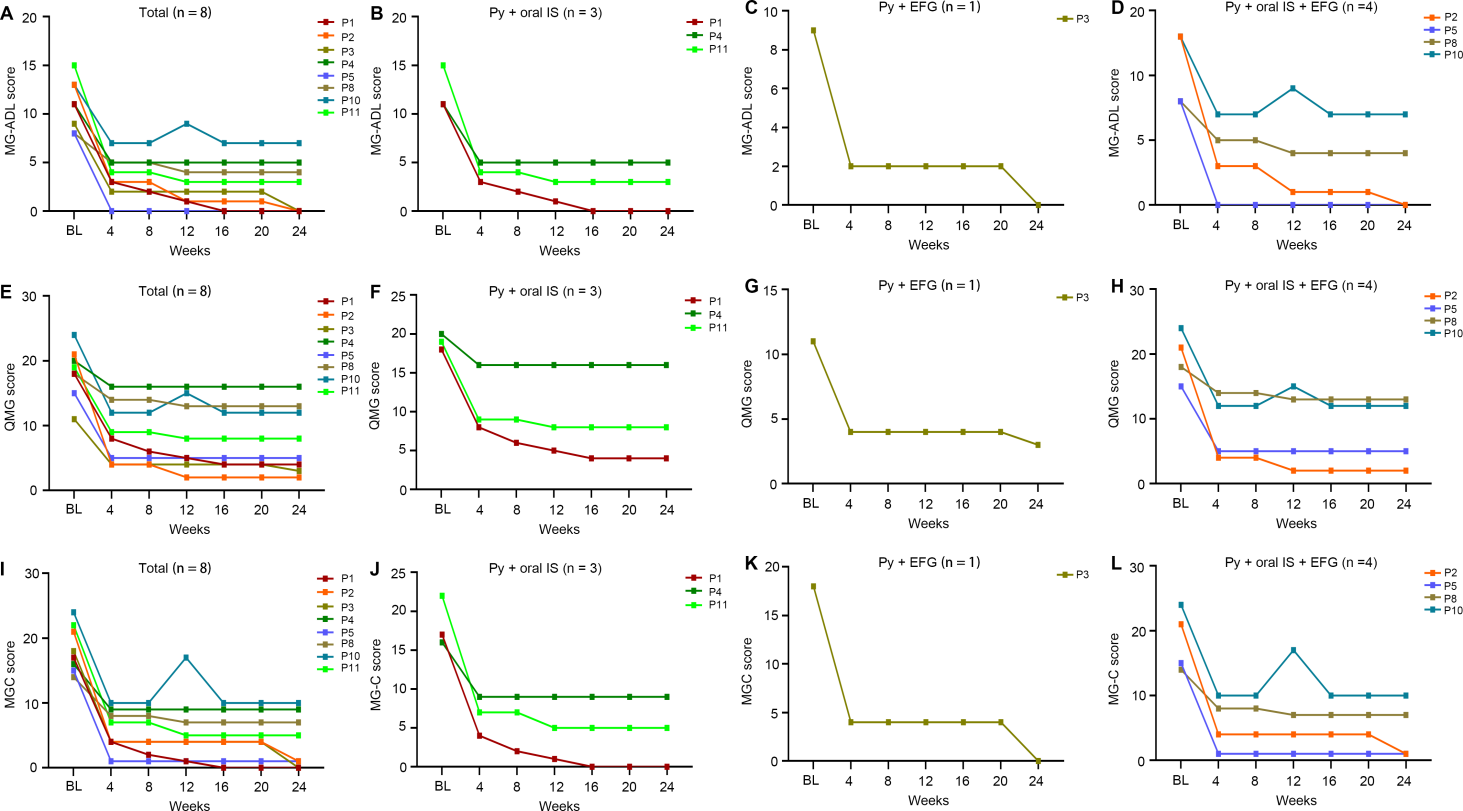
Longitudinal changes of clinical scores for the responded elderly gMG patients from baseline to the last visit time at week 24 and the patients were stratified by the sequential therapies after the first cycle of efgartigimod. **(A-D)** Detailed changes of MG-ADL for the responded elderly gMG patients; **(E-H)** Detailed changes of QMG for the responded elderly gMG patients; **(I-L)** Detailed changes of MGC for the responded elderly gMG patients; BL, baseline; EFG, efgartigimod; MG-ADL, Myasthenia Gravis-Activities of Daily Living; MGC, Myasthenia Gravis Composite; QMG, quantitative Myasthenia Gravis; Py, pyridostigmine; IS, immunosuppressant.

Following the initial efgartigimod treatment cycle, therapeutic management varied among patients. Five patients continued regular efgartigimod maintenance therapy (every 2–4 weeks); four patients received pyridostigmine plus oral immunosuppressants; one patient received pyridostigmine with efgartigimod; four patients were treated with pyridostigmine, oral immunosuppressants and efgartigimod; and the remaining three patients, who were non-responders, were transitioned to complement inhibitor therapy plus mycophenolate mofetil. During follow-up, one responder (P12) experienced symptom recurrence to baseline levels but declined further efgartigimod treatment and was lost to follow-up. Among the remaining eight responders, longitudinal assessments of MG-ADL (**Table 3**), QMG (**Supplementary Table S1**), and MGC (**Supplementary Table S2**) scores demonstrated sustained improvement throughout the 24-week observation period. The overall responders showed marked clinical progress, with mean MG-ADL, QMG, and MGC scores decreasing from 11.0, 18.3, and 18.3 at baseline to 2.4, 7.9, and 4.1 at week 24, respectively. This improvement was particularly evident in patients who initiated treatment during an MGAE (n=7). Furthermore, the single MUSK-Ab+ patient achieved complete remission (MG-ADL and MGC scores of 0) by the end of the study. These clinical benefits were maintained regardless of subsequent treatment regimen after the initial efgartigimod cycle (**Figures 4A–L**).

**Table 3:** The long-term efficacy profile of efgartigimod in 8 responded elderly gMG patients with age>80 years.

Group	Baseline MG-ADL (MSE%)	4w MG-ADL (MSE%)	8w MG-ADL (MSE%)	12w MG-ADL (MSE%)	16w MG-ADL (MSE%)	20w MG-ADL (MSE%)	24w MG-ADL (MSE%)
All patients (n=8)	11.0 ± 2.6 (0%)	3.6 ± 2.1 (12.5%)	3.5 ± 2.2 (12.5%)	3.1 ± 2.9 (37.5%)	2.8 ± 2.5 (37.5%)	2.8 ± 2.5 (37.5%)	2.4 ± 2.8 (50.0%)
AChR-Ab+ (n=7)	11.3 ± 2.6 (0%)	3.9 ± 2.2 (14.3%)	3.7 ± 2.3 (14.3%)	3.3 ± 3.1 (42.9%)	2.9 ± 2.7 (42.9%)	2.9 ± 2.7 (42.9%)	2.7 ± 2.8 (42.9%)
MUSK-Ab+ (n=1)	9.0 ± 0 (0%)	2.0 ± 0 (0%)	2.0 ± 0 (0%)	2.0 ± 0 (0%)	2.0 ± 0 (0%)	2.0 ± 0 (0%)	0.0 ± 0 (100%)
Initiation status							
MGAE (n=7)	11.4 ± 2.4 (0%)	3.4 ± 2.2 (14.3%)	3.3 ± 2.3 (14.3%)	3.0 ± 3.1 (42.9%)	2.6 ± 2.6 (42.9%)	2.6 ± 2.6 (42.9%)	2.1 ± 2.9 (57.1%)
mild/moderate (n=1)	6.7 ± 2.3 (0%)	5.3 ± 2.5 (0%)	5.3 ± 2.5 (0%)	5.0 ± 2.6 (0%)	4.3 ± 1.5 (0%)	4.3 ± 1.5 (0%)	4.0 ± 1.0 (0%)
Subsequent therapies							
Py + oral IS (n=3)	12.3 ± 2.3 (0%)	4.0 ± 1.0 (0%)	3.7 ± 1.5 (0%)	3.0 ± 2.0 (33.3%)	2.7 ± 2.5 (33.3%)	2.7 ± 2.5 (33.3%)	2.7 ± 2.5 (33.3%)
Py + EFG (n=1)	9.0 ± 0 (0%)	2.0 ± 0 (0%)	2.0 ± 0 (0%)	2.0 ± 0 (0%)	2.0 ± 0 (0%)	2.0 ± 0 (0%)	0.0 ± 0 (100%)
Py + oral IS + EFG (n=4)	10.5 ± 2.9 (0%)	3.8 ± 3.0 (25%)	3.8 ± 3.0 (25%)	3.5 ± 4.0 (50%)	3.0 ± 3.2 (50%)	3.0 ± 3.2 (50%)	2.8 ± 3.4 (50%)

EFG, efgartigimod; MG-ADL, Myasthenia Gravis-Activities of Daily Living; MSE, minimal symptom expression; MGAE, myasthenia gravis acute exacerbation; Py, pyridostigmine; IS, immunosuppressant.

**Figure 5:**
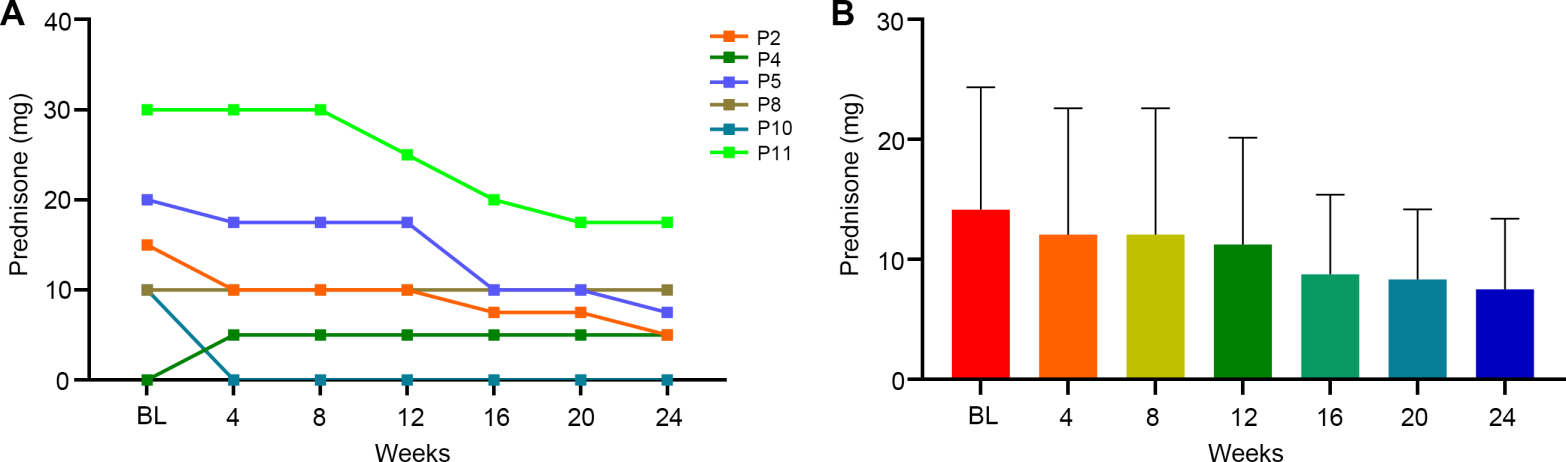
The effect of efgartigimod on prednisone doses. Six (6/8) efgartigimod responsed patients received prednisone treatment at the same time as efgartigimod initiated. The prednisone doses were recorded every 4 weeks until 24 weeks. **(A)** Detailed changes of prednisone for each of the 6 patients. Each line represented one patient. **(B)** Average change trend of the prednisone doses for the 6 patients. BL, baseline.

Among efgartigimod responders, 6/8 patients received prednisone during and after the initial treatment cycle. All baseline steroid users successfully reduced their daily prednisone dosage during the study period (**Figures 5A, B**). The average daily prednisone dose decreased by 2.1 mg per day at week 4, 2.9 mg at week 12, and 6.7 mg per day at week 24. By week 24, the mean daily prednisone dose was 7.5 mg, with 83.3% (5/6) of steroid-treated patients achieving maintenance doses ≤10 mg/day. One steroid-naïve patient initiated low-dose prednisone (5 mg/day) during therapy.

### 3.5 Patients without adequate rapid effect after efgartigimod treatment

In this study, one patient failed to achieve CMI by week 4, while two other patients were unable to sustain CMI through week 4. The clinical information of these cases are described in detail as follows.

**Figure 6:**
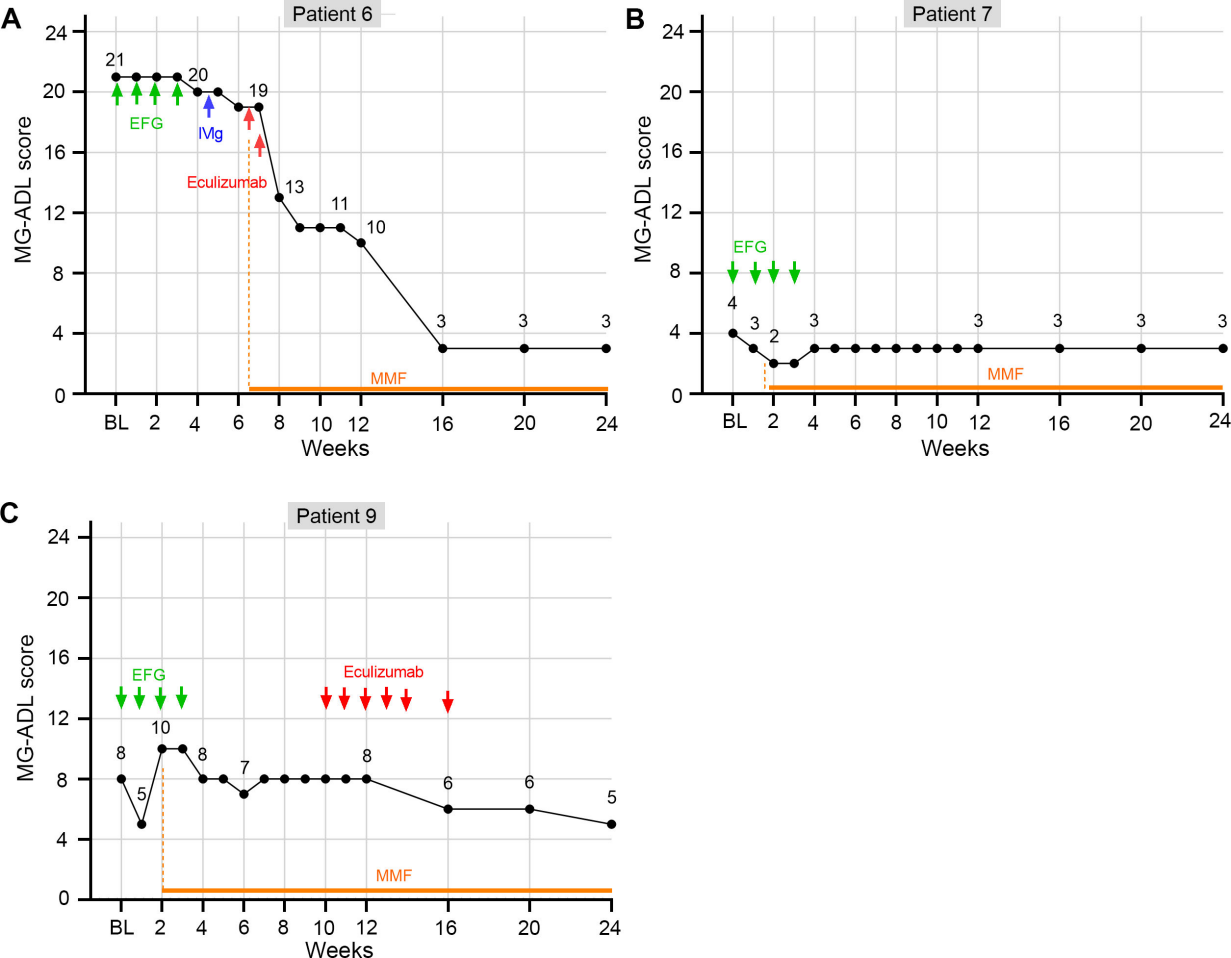
The changes of MG-ADL score of three elderly gMG patients who did not response quickly to efgartigimod within 4 weeks. **(A)** MG-ADL change trend for patient 6; **(B)** MG-ADL change trend for patient 7; **(C)** MG-ADL change trend for patient 9.

*Patient 6* (LOMG with acute deterioration): an 83-year-old female diagnosed with non-thymomatous AChR antibody-positive MG for two months. She only had ptosis of the eyelids and only took pyridostigmine when diagnosis. However, she developed MC and required intubation and mechanical ventilation at admission. Intravenous administration of efgartigimod was given from the second day (January 2024), however, only 1-point change of MG-ADL was achieved after receiving one cycle of efgartigimod treatment. Then, one cycle of IVIg (2g/kg over 5 days) with addition of oral mycophenolate mofetil from March 2024 continued without noticeable remission. Two doses of eculizumab (900 mg) were given at two and eight days after the IVIg treatment. The patient presented significant improvement and wean from ventilation at five days post the second dose of eculizumab infusion (week 8). By the latest visit at week 24, the patient’s clinical symptoms were still improving (**Figure 6A**).

*Patient 7* (Mild LOMG): an 82-year-old female, diagnosed with non-thymomatous AChR antibody-positive gMG for thirty months, complained with unsatisfied control of dysphagia and limb weakness (MGFA IIb, MG-ADL = 4, with 3 points in ptosis, and 1 point in the breath) at admission. She declined a daily dose increase of oral prednisolone (20 mg) as well as other immunosuppressants as additional treatment. So, one cycle of efgartigimod was applied. The score of MG-ADL decreased to 2 (with 2 points in ptosis) at weeks 2 and 3. However, 1-point increase in the breath was recorded at week 4, resulting in the total MG-ADL score of 3 (**Figure 6B**). No improvement was recorded during the follow-up visit.

*Patient 9* (Moderate LOMG): an 83-year-old female, diagnosed with non-thymomatous AChR antibody-positive gMG for three months, and presented with acute worsening in limbs for one-week (MGFA IIIa, MG-ADL = 8, with 1 points in speaking, 1 point in chewing, 1 point in swallowing, 1 point in breath, 2 points in diplopia, and 2 points in ptosis). After receiving the first infusion of efgartigimod, CMI was achieved at week 1, with MG-ADL reduced to 5. However, the diplopia and ptosis exacerbated again at weeks 2 and 3 (both MG-ADL = 10). At week 4, one week after the last infusion of efgartigimod, the clinical symptoms returned to the baseline levels (MG-ADL = 8). Then, the patient switched to eculizumab treatment and MG-ADL was reduced to 5 at the last visit at week 24 (**Figure 6C**).

### 3.6 Safety of efgartigimod in the elderly patients

Three patients experienced side effects. Two patients complained of transient minor headache for one day after each infusion. One patient had upper respiratory tract infection which was resolved by antimicrobials. These side effects did not lead to efgartigimod discontinuation. Other patients tolerated the efgartigimod treatment well without any documented side effects. The overall TEAE incidence rate was 25% over the 24 weeks.

## 4 Discussion

Elderly gMG patients were substantially underrepresented in both the ADAPT trial and subsequent real-world studies. Our multicenter retrospective study addresses this gap by providing clinical evidence supporting efgartigimod use in elderly gMG patients (mean age: 82.9 ± 2.5 years), while evaluating efficacy across antibody subtypes and baseline disease severity.

The patients in our cohort have the representative clinical characteristics of VLOMG. Consistent with previous reports (3, 5, 11), all patients presented disease onset after 65 years of age (mean age: 81.3 ± 4.1 years), none had thymoma, and most were anti-AChR antibody positive. Interestingly, our cohort showed a female predominance, contrasting with the male predominance in some VLOMG studies. This discrepancy may be resolved with larger sample sizes. Notably, all patients presented with multiple comorbidities (≥2 conditions per patient), and the majority (11/12) had some degree of renal impairment, complicating MG management in this elderly population.

Despite the challenges of managing MG in elderly patients, our findings demonstrated that efgartigimod provided rapid disease control even in octogenarians, suggesting its potential as an effective therapeutic option for this vulnerable population. If assessed by the proportion of MG patients achieving CMI after one cycle of efgartigimod treatment, the therapeutic response to efgartigimod in the present study was even better than the younger patients reported in the ADAPT trial and a US multicenter cohort (91.7%, 68%, and 72%, respectively) (12, 18). However, the proportion of patients to achieve MSE after one cycle of efgartigimod treatment was the lowest in the present study when compared to the other two studies (8.3% *vs*. 40% and 25%). The average time to achieve CMI was slightly longer than that of the LOMG reported in a Chinese multicentric study (1.3 ± 0.5 weeks *vs*. 1.1 ± 0.4) (17). Besides age differences, these less favorable outcomes might be attributed to the more severe disease status of patients included in the present study, as reflected by higher baseline MG-ADL scores. Although the proportion of patients to achieve MSE was not satisfactory, it kept increasing during the follow-up period, with four patients achieving MSE by week 24. This trend aligns with the LOMG cases in the Chinese cohort study, whereas younger patients in the ADAPT trial and the Chinese cohort experienced symptom rebound during follow-up (12, 17). Previous research has suggested that different responses to efgartigimod between EOMG and LOMG may be related to the profound effect of thymic inflammation on immunopathogenesis (17). Thymic follicular hyperplasia occurs often in the EOMG and associates with the intrathymic production of IgG autoantibodies against AChR, whereas thymic atrophy is characteristic of LOMG (19, 20). Additionally, the concurrent or subsequent use of oral immunosuppressants, combined with age-related alterations in renal and hepatic clearance affecting drug half-life, may also contribute to these differences (21).

Although efgartigimod is approved for the treatment of AChR-antibody positive MG, growing evidence indicated that it was also well-tolerated and efficacious in MuSK-antibody positive and triple-negative gMG patients (17, 22–24). This study also revealed favorable effects of efgartigimod in treating elderly MuSK-antibody positive MG patients. The disease mechanism of MuSK-MG was significantly different from that of AChR-MG (25). The subclass of autoantibodies against AChR are predominantly IgG1 and IgG3, which impair neuromuscular transmission by activation of complement, cross-linking and degradation of AChR by internalization, and completion for binding of ACh. MuSK autoantibodies are predominantly IgG4 subclass, which works by obstructing the protein-protein interaction at neuromuscular junctions. Despite these differences, efgartigimod has been proven to clear pathogenic IgG1, IgG3 and IgG4 with comparable efficiency (26). It is worth noting that the limited number of elderly MuSK-MG cases in the present study precluded definite conclusions. Future study with large number of MuSK-MG are needed to verify these findings.

Recent studies have validated the rapid action of efgartigimod in controlling symptom progression in myasthenic crisis (15, 16). In this study, we also stratified patients by disease activity at treatment initiation: eight MGAE, three mild/moderate, and one MC case. Efgartigimod served as an effective fast-acting therapy for elderly patients in MGAE status. However, mild/moderate cases exhibited less favorable responses, with two out of the three patients showed no or slight MG-ADL reduction after efgartigimod treatment. We hypothesize that patients with milder disease may have a less inflammatory or autoantibody-driven pathophysiology, or may have different expectations regarding treatment goals. Significant improvement of the MC case was exhibited after the addition of IVIg and C5 inhibitor. This suggested that incorporating biologics with different targets might be an effective and safe strategy for treating elderly MC patients. Nonetheless, the small number of MC (n =1) and mild/moderate (n =3) cases precludes definite conclusions. As younger patients in MC and mild/moderate status have shown positive response to efgartigimod (17), further studies are needed to explore whether disease severity at treatment initiation influences efgartigimod response in the elderly.

Efgartigimod has increased affinity to FcRn and outcompetes endogenous IgG binding, thereby reducing IgG recycling and increasing IgG degradation (26). In the ADAPT and ADAPT+ trials, patients had serum IgG levels less than 6 g/L were excluded at screening (12, 24). The suitability of efgartigimod for patients with baseline IgG <6 g/L remains unclear. One of our patients with baseline IgG level of 4.24 g/L received one cycle of efgartigimod safely. The maximum IgG reduction (59.0%, from 4.24 g/L to 1.74 g/L) and best clinical improvement occurred at 1 week after the second infusion. However, continued efgartigimod administration did not yield further IgG reduction or symptom improvement. In previous reports, the mean maximum reduction of IgG in AChR antibody-positive patients (61.3% ± 0.9%) was achieved one week after the fourth infusion of efgartigimod, and the classical dosing regimen of efgartitimod was four weekly infusions as one cycle (12, 24). The discrepancy indicated that for patients with low IgG baseline levels, the time to achieve maximum IgG reduction needed further exploration. Routine monitoring of IgG levels might be necessary to guide personalized and cost-effective use of efgartigimod.

This study has the advantage of providing new evidence for the efgartigimod application in elderly MG patients, especially for those over 80. Most previous studies describing real-world efgartigimod experience stratified participants by autoantibody profile. One multicenter real-world cohort explored responses in EOMG and LOMG but did not detail outcomes in the VLOMG (17). A recently published study included 15 VLOMG patients and reported better CMI and MSE rates after efgartigimod than the present study, but it only contained 2 patients over the age of 80 (27). Given improved diagnosis, treatment, and increasing human longevity, focusing on MG treatment in the elderly, even those over 80, is clinically meaningful (3, 11).

In MG management, early steroid dose reduction to minimize side effects is crucial. However, determining the optimal timing and rate for steroid tapering is particularly challenging and requires considerable caution in elderly patients. In our study, steroid tapering was individualized based on clinical response and comorbidities. The majority of patients on prednisone achieved a maintenance dose of ≤10 mg/day by week 24. This suggests that efgartigimod may facilitate steroid sparing in this vulnerable population, though the optimal tapering schedule remains to be established in future studies.

There are several limitations in this study. Although we aimed to include all consecutive eligible patients aged ≥80 years from the participating centers, the retrospective nature of this study may have introduced selection bias. For instance, patients with more severe disease or those receiving novel therapies might have been more systematically documented. The absence of complete serological data, particularly for IgG and antibody titers, limited our ability to fully correlate immunological changes with clinical outcomes. The consistent collection of QMG and MGC scores at weekly intervals during the first cycle (Weeks 1, 2, 3) was not feasible across all centers. Differences in genetic background, healthcare access, and treatment practices could influence the efficacy and safety profile of efgartigimod in elderly MG patients outside China. As our study included only Chinese patients, the generalizability of our findings to other ethnic or geographic populations may be limited. The lack of a control group prevents direct comparison with standard therapies, and the relatively short follow-up period restricts assessment of long-term efficacy and safety. Furthermore, the small sample size and absence of SNMG cases mean that the results should be interpreted cautiously. Multinational studies or a larger prospective controlled study with long-term follow-up period are needed to verify our conclusions.

## 5 Conclusion

In conclusion, our findings establish efgartigimod as a well-tolerated and clinically effective treatment option for generalized myasthenia gravis in patients aged 80 years and older - a population with high unmet therapeutic needs. Its rapid onset of action and steroid-sparing potential are particularly valuable in this vulnerable, multimorbid age group.

## Data Availability

The original contributions presented in the study are included in the article/**Supplementary Material**. Further inquiries can be directed to the corresponding authors.

## Glossary

AChR: acetylcholine receptor
CMI: clinical meaningful improvement
EOMG: early-onset myasthenia gravis
FcRn: neonatal Fc receptor
EFG: efgartigimod
gMG: generalized myasthenia gravis
LOMG: late-onset myasthenia gravis
MG: myasthenia gravis
MG-ADL: Myasthenia Gravis Activities of Daily Living
MGAE: myasthenia gravis acute exacerbation
MGC: Myasthenia Gravis Composite score
MC: myasthenic crisis
MGFA: Myasthenia Gravis Foundation of America
MuSK: muscle-specific tyrosine kinase
MSE: minimal symptom expression
QMG: quantitative Myasthenia Gravis
VLOMG: very-late-onset myasthenia gravis
SNMG: seronegative myasthenia gravis
TAE: treatment adverse effects
TMG: thymoma-associated myasthenia gravis

## References

[1] Gilhus NE Tzartos S Evoli A Palace J Burns TM Verschuuren JJGM . Myasthenia gravis. Nat Rev Dis Primers. (2019) 5:30. doi: 10.1038/s41572-019-0079-y, PMID: 31048702

[2] Huijbers MG Marx A Plomp JJ Panse RL Phillips WD . Advances in the understanding of disease mechanisms of autoimmune neuromuscular junction disorders. Lancet Neurol. (2022) 21:163–75. doi: 10.1016/S1474-4422(21)00357-4, PMID: 35065039

[3] Cortés-Vicente E Lvarez-Velasco R Segovia S Paradas C Illa I . Clinical and therapeutic features of myasthenia gravis in adults based on age at onset. Neurology. (2020) 94:e1171–80. doi: 10.1212/WNL.0000000000008903, PMID: 32071167 PMC7220233

[4] Nielsen JJJ Levison L Andersen H . Changes in myasthenia gravis incidence in Denmark from 1985 to 2021:A Nationwide Population-Based Study. Neurology. (2024) 104(1):e210139. doi: 10.1212/WNL.0000000000210139, PMID: 39666916

[5] Tang YL Ruan Z Su Y Guo R-J Gao T Liu Yu . Clinical characteristics and prognosis of very late-onset myasthenia gravis in China. Neuromuscular disorders: NMD. (2023) 33:358–66. doi: 10.1016/j.nmd.2023.02.013, PMID: 36990040

[6] Bruckman D Lee I Schold JD Claytor BR Silvestri NJ Hehir MK . Epidemiologic study of myasthenia gravis in the elderly US population: A longitudinal analysis of the medicare claims database, 2006-2019. Neurology. (2024) 103:e210005. doi: 10.1212/WNL.0000000000210005, PMID: 39496108 PMC11540456

[7] Sanders DB Wolfe GI Benatar M Evoli A Narayanaswami P . International consensus guidance for management of myasthenia gravis: Executive summary. Neurology. (2016) 87:419–25. doi: 10.1212/WNL.0000000000002790, PMID: 27358333 PMC4977114

[8] Nelke C Stascheit F Eckert C Pawlitzki M Schroeter CB Huntemann N . Independent risk factors for myasthenic crisis and disease exacerbation in a retrospective cohort of myasthenia gravis patients. J Neuroinflamm. (2022) 19:1–12. doi: 10.1186/s12974-022-02448-4, PMID: 35413850 PMC9005160

[9] Chanson JB Bouhour F Aubé-Nathier AC Mallaret M Vial C Hacquard A . Myasthenia gravis treatment in the elderly presents with a significant iatrogenic risk: a multicentric retrospective study. J Neurol. (2023) 270:5819–26. doi: 10.1007/s00415-023-11925-6, PMID: 37592137

[10] Romi F Gilhus NE Varhaug JE Myking A Aarli JA . Thymectomy and anti-muscle autoantibodies in late-onset myasthenia gravis. Eur J Neurol. (2002) 9:55–61. doi: 10.1046/j.1468-1331.2002.00352.x, PMID: 11784377

[11] Aarli JA . Myasthenia gravis in the elderly: Is it different? Ann New York Acad Sci. (2008) 1132:238–43. doi: 10.1196/annals.1405.040, PMID: 18567874

[12] Howard JF Jr. Bril V Vu T Karam C Peric S Margania T . Safety, efficacy, and tolerability of efgartigimod in patients with generalised myasthenia gravis (ADAPT): a multicentre, randomised, placebo-controlled, phase 3 trial. Lancet Neurol. (2021) 20:526–36. doi: 10.1016/S1474-4422(21)00159-9, PMID: 34146511

[13] Suzuki S Uzawa A Nagane Y Masuda M Konno S Kubota T . Therapeutic responses to efgartigimod for generalized myasthenia gravis in Japan. Neurol Clin Pract. (2024) 14:e200276. doi: 10.1212/CPJ.0000000000200276, PMID: 38544885 PMC10965358

[14] Fuchs L Shelly S Vigiser I Kolb H Regev K Schwartzmann Y . Real-World experience with efgartigimod in patients with myasthenia gravis. J Neurol. (2024) 271:3462–70. doi: 10.1007/s00415-024-12293-5, PMID: 38528163

[15] Hong Y Gao L Huang S-Q Liu S Feng S Chen Y-B . Efgartigimod as a fast-acting add-on therapy in manifest and impending myasthenic crisis: A single-center case series. J Neuroimmunol. (2024) 395:578431. doi: 10.1016/j.jneuroim.2024.578431, PMID: 39142025

[16] Song J Wang H Huan X Jiang Q Wu Z Yan C . Efgartigimod as a promising add-on therapy for myasthenic crisis: a prospective case series. Front Immunol. (2024) 15:1418503. doi: 10.3389/fimmu.2024.1418503, PMID: 39136012 PMC11317420

[17] Luo S Jiang Q Zeng W Wang Q Zou Z Yu Y . Efgartigimod for generalized myasthenia gravis: A multicenter real-world cohort study in China. Ann Clin Trans Neurol. (2024) 11:2212–21. doi: 10.1002/acn3.52142, PMID: 38973109 PMC11330228

[18] Katyal N Halldorsdottir K Govindarajan R Shieh P Muley S Reyes P . Safety and outcomes with efgartigimod use for acetylcholine receptor-positive generalized myasthenia gravis in clinical practice. Muscle Nerve. (2023) 68:762–6. doi: 10.1002/mus.27974, PMID: 37695277

[19] Donaldson DH Ansher M Horan S Rutherford RB Ringel SP . The relationship of age to outcome in myasthenia gravis. Neurology. (1990) 40:786–90. doi: 10.1212/WNL.40.5.786, PMID: 2330105

[20] Wolfe GI Kaminski HJ Aban IB Minisman G Kuo HC Marx A . Randomized trial of thymectomy in myasthenia gravis. New Engl J Med. (2016) 375:511–22. doi: 10.1056/NEJMoa1602489, PMID: 27509100 PMC5189669

[21] Mangoni AA Jackson SH . Age-related changes in pharmacokinetics and pharmacodynamics: basic principles and practical applications. Br J Clin Pharmacol. (2004) 57:6–14. doi: 10.1046/j.1365-2125.2003.02007.x, PMID: 14678335 PMC1884408

[22] Frangiamore R Rinaldi E Vanoli F Andreetta F Mantegazza R Antozzi C . Efgartigimod improves triple-negative myasthenia gravis. Neurological Sci. (2024) 45:1307–9. doi: 10.1007/s10072-023-07122-y, PMID: 37875596

[23] Singer M Khella S Bird S Mcintosh P Paudyal B Wadhwani A . Single institution experience with efgartigimod in patients with myasthenia gravis: Patient selection, dosing schedules, treatment response, and adverse events. Muscle Nerve. (2024) 69:87–92. doi: 10.1002/mus.28003, PMID: 37990374

[24] Howard JF Bril V Vu T Karam C Peric S De Bleecker JL . Long-term safety, tolerability, and efficacy of efgartigimod (ADAPT): interim results from a phase 3 open-label extension study in participants with generalized myasthenia gravis. Front Neurol. (2024) 14:1284444. doi: 10.3389/fneur.2023.1284444, PMID: 38318236 PMC10842202

[25] Tannemaat MR Huijbers MG Verschuuren JJGM . Myasthenia gravis-Pathophysiology, diagnosis, and treatment. Handb Clin Neurol. (2024) 200:283–305. doi: 0.1016/B978-0-12-823912-4.00026-8, PMID: 38494283 10.1016/B978-0-12-823912-4.00026-8

[26] Ulrichts P Guglietta A Dreier T van Bragt T Hanssens V Hofman E . Neonatal Fc receptor antagonist efgartigimod safely and sustainably reduces IgGs in humans. J Clin Invest. (2018) 128:4372–86. doi: 10.1172/JCI97911, PMID: 30040076 PMC6159959

[27] Zhang Z Yang M Guo X Ma T Wang Z Luo T . Efgartigimod as a fast-acting treatment in generalized very-late-onset myasthenia gravis. Front Immunol. (2025) 16:1579859. doi: 10.3389/fimmu.2025.1579859, PMID: 40313945 PMC12043626

