# Supplementary material for: Efgartigimod for generalized myasthenia gravis in the extreme elderly (≥80 years): a multicenter retrospective real-world study
Source: Front Immunol. 2025 Nov 6;16:1685233. doi: 10.3389/fimmu.2025.1685233 (PMC12631306; doi:10.3389/fimmu.2025.1685233)
Supplement: Supplementary file 1 [file DataSheet1.pdf]

Table S1. The long-term efficacy reflected by QMG change in 8 efgartigmod-responded elderly gMG patients with age > 80 years.

Group	Baseline QMG	4w QMG	8w QMG	12w QMG	16w QMG	20w QMG	24w QMG
All patients (n=8)	18.3 ± 3.9	9.0 ± 4.6	8.8 ± 4.7	8.5 ± 5.4	8.0 ± 5.1	8.0 ± 5.1	7.9 ± 5.2
AChR-Ab+ (n=7)	19.3 ± 2.8	9.7 ± 4.5	9.4 ± 4.7	9.1 ± 5.5	8.6 ± 5.2	8.6 ± 5.2	8.6 ± 5.2
MUSK-Ab+ (n=1)	11.0 ± 0	4.0 ± 0	4.0 ± 0	4.0 ± 0	4.0 ± 0	4.0 ± 0	3.0 ± 0
Initiation status							
MGAE (n=7)	18.3 ± 4.2	8.3 ± 4.5	8.0 ± 4.6	7.9 ± 5.5	7.3 ± 5.1	7.3 ± 5.1	7.1 ± 5.2
mild/moderate (n=1)	18.0 ± 0	14.0 ± 0	14.0 ± 0	13.0 ± 0	13.0 ± 0	13.0 ± 0	13.0 ± 0
Subsequent therapies							
Py + oral IS (n=3)	19.0 ± 1.0	11.0 ± 4.4	10.3 ± 5.1	9.7 ± 5.7	9.3 ± 6.1	9.3 ± 6.1	9.3 ± 6.1
Py + EFG (n=1)	11.0 ± 0	4.0 ± 0	4.0 ± 0	4.0 ± 0	4.0 ± 0	4.0 ± 0	3.0 ± 0
Py + oral IS + EFG (n=4)	19.5 ± 3.9	8.8 ± 5.0	8.8 ± 5.0	8.8 ± 5.0	8.0 ± 5.4	8.0 ± 5.4	8.0 ± 5.4

EFG: efgartigimod; QMG, quantitative Myasthenia Gravis score; MGAE, myasthenia gravis acute exacerbation;Py: pyridostigmine; IS: immunosuppressant.

Table S2. The long-term efficacy reflected by MGC change in 8 efgartigmod-responded gMG patients with age > 80 years.

Group	Baseline MGC	4w MGC	8w MGC	12w MGC	16w MGC	20w MGC	24w MGC
All patients (n=8)	18.3 ± 3.6	5.9 ± 3.1	5.6 ± 3.3	6.0 ± 5.2	5.0 ± 3.5	5.0 ± 3.5	4.1 ± 4.1
AChR-Ab+ (n=7)	18.4 ± 3.9	6.1 ± 3.2	5.9 ± 3.5	6.3 ± 5.6	5.1 ± 3.8	5.1 ± 3.8	4.7 ± 4.1
MUSK-Ab+ (n=1)	18.0 ± 0	4.0 ± 0	4.0 ± 0	4.0 ± 0	4.0 ± 0	4.0 ± 0	0.0 ± 0
Initiation status							
MGAE (n=7)	19.0 ± 3.4	5.6 ± 3.2	5.3 ± 3.5	5.9 ± 5.6	4.7 ± 3.7	4.7 ± 3.7	3.7 ± 4.3
mild/moderate (n=1)	14.0 ± 0	8.0 ± 0	8.0 ± 0	7.0 ± 0	7.0 ± 0	7.0 ± 0	7.0 ± 0
Subsequent therapies							
Py + oral IS (n=3)	18.3 ± 3.2	6.7 ± 2.5	6.0 ± 3.6	5.0 ± 4.0	4.7±4.5	4.7± 4.5	4.7± 4.5
Py + EFG (n=1)	18.0 ± 0	4.0 ± 0	4.0 ± 0	4.0 ± 0	4.0 ± 0	4.0 ± 0	0.0 ± 0
Py + oral IS + EFG (n=4)	18.5 ± 4.8	5.8 ± 4.0	5.8 ± 4.0	7.3 ± 6.9	5.5 ± 3.9	5.5 ± 3.9	4.8 ± 4.5

EFG: efgartigimod; MGC,Myasthenia Gravis Composite ; MGAE, myasthenia gravis acute exacerbation;Py: pyridostigmine; IS: immunosuppressant.
